# Multi-Kingdom Synergy of *Perilla frutescens*-Derived Effector Vesicles and Postbiotics: A Triple-Action Strategy for Atopic Dermatitis

**DOI:** 10.3390/life16050769

**Published:** 2026-05-04

**Authors:** Andrea Badale, Mihaela Zdrîncă, Laura Maghiar, Ioan Magyar, Dana Carmen Zaha

**Affiliations:** 1 Department of Preclinical Disciplines, Discipline of Pharmacology, Faculty of Medicine and Pharmacy, University of Oradea, 410087 Oradea, Romania; mzdrinca@yahoo.com (M.Z.); magyar_nelu@yahoo.com (I.M.); 2 Department of Psychoneurosciences and Rehabilitation, Faculty of Medicine and Pharmacy, University of Oradea, 410087 Oradea, Romania; lauratodan@yahoo.ro; 3 Department of Preclinical Disciplines, Discipline of Physiology, Faculty of Medicine and Pharmacy, University of Oradea, 410087 Oradea, Romania; danaczaha@gmail.com

**Keywords:** AD, skin microbiome, postbiotics, *Lactobacillus*, *Saccharomyces*, *Perilla frutescens*, plant vesicles, JAK–STAT pathway, skin barrier restoration

## Abstract

Background: Atopic dermatitis (AD) is a chronic inflammatory disease characterized by profound microbial dysbiosis, *Staphylococcus aureus* (*S. aureus*) colonization, and a compromised epidermal barrier. Current therapies often face safety and compliance limitations, necessitating proactive, steroid-sparing ecological strategies focused on barrier restoration. Methods: This pharmacological review evaluates a synergistic framework combining *Lactobacillus* and *Saccharomyces* postbiotic lysates with *Perilla frutescens*-derived effector vesicles. The analysis focuses on their molecular impact on skin homeostasis and structural protein synthesis. Results: In vitro evaluations indicate that *Lactobacillus* enhances innate immunity, while *Saccharomyces*-derived metabolites support the microbial ecosystem. Preliminary data demonstrate a significant impact on structural integrity, showing an 87.9% increase in elastin secretion and a 61.4% increase in Type I collagen synthesis. Furthermore, *Perilla frutescens*-derived vesicles modulate the JAK–STAT pathway, demonstrating a potential reduction of Interleukin-6 (IL-6) by 40% and a downregulation of CYP1A1 expression by up to 49% in cell models, thereby suggesting a capacity to mitigate oxidative stress and pruritus. Conclusions: By integrating these components into a “Triple-Action” framework, focusing on immunomodulation, structural restoration, and precision signaling, this review provides a comprehensive roadmap for promising dermo-cosmetic interventions in atopic skin management.

## 1. Introduction

AD is a chronic, relapsing inflammatory skin disease that imposes a significant global burden, characterized by profound skin barrier dysfunction and complex immune dysregulation [1]. The pathogenesis of AD is multifaceted, involving a delicate interplay between genetic predisposition, environmental triggers, and a persistent state of cutaneous inflammation mediated by Th2-polarized responses. Recent clinical evidence highlights that the integrity of the epidermal barrier is not merely a structural concern but is deeply intertwined with the ecological balance of the skin microbiome [2].

The skin of AD patients frequently exhibits a state of dysbiosis, characterized by a significant reduction in microbial diversity. Beyond the well-documented overcolonization by *S. aureus*, recent research emphasizes the critical role of the loss of protective commensals, such as *Staphylococcus epidermidis (S. epidermidis)* and *Staphylococcus hominis* (*S. hominis*). These beneficial species are essential for maintaining homeostasis by inducing host antimicrobial peptides (AMPs) and inhibiting pathogen growth. Notably, the degree of microbial dysbiosis and the decline in commensal diversity have been directly correlated with disease severity, increased pruritus, and the extent of barrier dysfunction [2]. *S. aureus* further exacerbates this inflammatory cycle by secreting toxins and proteases that degrade the physical barrier and trigger pro-inflammatory signaling pathways [2]. Conventional therapeutic strategies, predominantly focused on topical corticosteroids and immunosuppressants, often provide symptomatic relief but may lead to long-term side effects, such as skin atrophy, necessitating the exploration of safer, adjunctive, or proactive therapies [3,4].

In this context, the modulation of the skin microbiome has emerged as a promising frontier. While live probiotics face challenges regarding stability and safety in topical formulations, postbiotics—defined by the International Scientific Association for Probiotics and Prebiotics (ISAPP) as “preparations of inanimate microorganisms and/or their components that confer a health benefit”—offer a viable alternative [5,6]. These cell-free lysates and metabolic by-products, derived from strains such as *Lactobacillus* and *Saccharomyces*, possess immunomodulatory, antimicrobial, and barrier-restoring properties [2,7,8].

The integration of advanced biotechnologies has led to the exploration of plant-derived nanovesicles (PDNVs) as biomimetic delivery systems and bioactive agents. Unlike synthetic nanoparticles, these plant-derived vesicles are highly biocompatible and can effectively penetrate the stratum corneum to deliver molecular cargo to deeper epidermal layers [9,10]. Specifically, *Perilla frutescens* has gained attention for its anti-allergic and anti-inflammatory properties; its bioactive compounds, such as rosmarinic acid and luteolin, modulate key signaling pathways, including the JAK–STAT and TLR-2 axes, which are often overactivated in atopic skin [11,12,13].

The synergistic use of microbial postbiotics and plant-derived nanovesicles represents a dual-strategy for restoring the skin’s physical and chemical barriers while promoting eubiosis. This combination aims to enhance the expression of structural proteins and provide a multi-dimensional approach to modulating the cutaneous immune response [14,15,16]. Emerging research indicates that specific metabolites from *Lactobacillus* and *Saccharomyces* ferments can enhance filaggrin expression and suppress oxidative stress, providing a holistic approach to skin health [17,18,19].

The aim of this review is to provide a comprehensive analysis of the synergistic potential between microbial lysates and *Perilla*-derived vesicles in the management of AD. We evaluate the underlying molecular mechanisms—ranging from Quorum Quenching to the inhibition of pro-inflammatory cytokines—and discuss how this targeted postbiotic approach may facilitate a “steroid-sparing” therapy that restores both the structural and ecological homeostasis of the skin [20].

## 2. Methodology and Review Approach

The literature search for this pharmacological review was conducted across high-impact databases, including PubMed/MEDLINE, Scopus, and Web of Science. To ensure an up-to-date perspective on the rapidly evolving fields of postbiotics and exosome research, the search strategy prioritized peer-reviewed articles and technical reports published between 2019 and 2026.

Keywords and Search Terms:

A combination of Boolean operators and the following keywords was utilized: “atopic dermatitis”, “skin microbiome dysbiosis”, “postbiotics”, “Lactobacillus lysates”, “Saccharomyces ferments”, “plant-derived nanovesicles”, and “Perilla frutescens exosomes”.

Inclusion and Exclusion Criteria:

- Inclusion: Recent studies (past 7 years) describing molecular mechanisms of skin barrier restoration, immunomodulatory effects of microbial lysates, and the biophysical characterization of plant vesicles.
- Exclusion: Older literature (prior to 2019, unless citing fundamental concepts), conference abstracts without full-text availability, and studies lacking specific mechanistic insights into cutaneous homeostasis.

This approach ensures that the synthesis integrates the latest advancements in dermo-cosmetic biotechnology with preclinical laboratory evaluations.

## 3. Comparative Pharmacology of Microbial Lysates

The pharmacological landscape of AD is currently witnessing a paradigm shift from traditional immunosuppression to microbiome-targeted interventions. At the forefront of this evolution are postbiotics, which, according to the consensus definition provided by ISAPP, are preparations of inanimate microorganisms and/or their components that confer a health benefit on the host [5,21]. Unlike live probiotics, postbiotics offer enhanced stability, precise dosing, and a superior safety profile, making them ideal for compromised skin barriers. In AD, the comparative pharmacology of *Lactobacillus* and *Saccharomyces* lysates reveals a biomimetic “multi-kingdom” synergy: while bacterial fractions specialize in immune priming and pathogen exclusion, fungal ferments provide the metabolic fuel and structural building blocks required for holistic epidermal reconstruction [3,22,23].

The comparative pharmacological profile and molecular targets of these postbiotics are summarized in Table 1.

### 3.1. Structural Biochemistry: Peptidoglycans vs. β-Glucans

The therapeutic efficacy of a postbiotic is fundamentally dictated by its cell wall architecture and the precision of the cellular disruption process. Bacterial lysates from the *Lactobacillus* genus (specifically *Lactiplantibacillus plantarum*) are defined by a high concentration of peptidoglycans (PGN) and lipoteichoic acids (LTA). These molecules act as high-affinity ligands for Pattern Recognition Receptors (PRRs), notably Toll-like Receptor 2 (TLR-2). The pharmacological activation of TLR-2 on keratinocytes triggers a complex intracellular signaling cascade via the NF-κB and MAPK pathways, which in turn upregulates the production of endogenous antimicrobial peptides (AMPs), such as human β-defensin 2 and cathelicidin LL-37. This “immune training” is vital for correcting the inherent immunological deficits found in AD patients [6,24]. However, the bioavailability of these ligands depends heavily on the lysis method employed. Mechanical disruption (such as high-pressure homogenization or sonication) often yields a more diverse array of immunostimulatory fragments compared to simple heat inactivation [43]. In contrast, the *Saccharomyces cerevisiae* cell wall is a multi-layered matrix dominated by β-1,3/1,6-glucans and mannoproteins. These fungal polysaccharides interact with Dectin-1 receptors, which are crucial for modulating the Th2-biased inflammatory response typical of AD and promoting deep tissue repair [25,26]. The bioavailability of these bioactives is strictly dependent on the cellular disruption process; while mechanical or enzymatic methods are essential to release these ‘trapped’ polysaccharides from the fungal matrix, excessive processing can lead to the denaturation of delicate carbohydrate structures [43,44]. Therefore, the standardization of manufacturing protocols—balancing lysis intensity with the preservation of structural integrity—is a fundamental requirement for ensuring the reproducible pharmacological activity of yeast-derived postbiotics [7,38].

### 3.2. Chemical and Biophysical Characterization of the PEC

To ensure the reproducibility of the mechanistic effects described in this study, the *Perilla* Effector Complex (PEC) was characterized in terms of its primary bioactive components and physical properties.

- Exosomal Fraction: The plant-derived effector vesicles (EVs) from *Perilla frutescens* were isolated and analyzed using Nanoparticle Tracking Analysis (NTA). The vesicles exhibited a typical exosomal size distribution, with a mean diameter ranging between 50 and 200 nm, consistent with stable lipid-bilayer vesicles capable of deep epidermal penetration [33].
- Postbiotic Profile: The fermented fraction, derived from the symbiotic action of *Lactobacillus* and *Saccharomyces*, was found to be rich in bioactive peptides, organic acids (specifically lactic acid), and β-glucans. These metabolites contribute to the acidification of the skin surface (pH regulation) and provide the metabolic substrates required for keratinocyte differentiation [38,44].
- Synergistic Formulation: The PEC was stabilized in an O/W emulsion, optimized for the sustained release of both the exosomal cargo and the postbiotic metabolites, ensuring high bioavailability at the dermal-epidermal junction [7].

### 3.3. Metabolic Profiles: From Acidification to Aryl Hydrocarbon Receptor (AhR) Activation

The “metabolic legacy” left by these microorganisms during controlled fermentation provides a secondary layer of pharmacological activity. *Lactobacillus* fermentation is synonymous with the production of lactic acid, a natural alpha-hydroxy acid (AHA) that serves two critical functions: it restores the “acid mantle” (maintaining a skin pH of 4.5–5.5) and acts as a potent humectant that stimulates ceramide synthesis [28,29]. A low surface pH is essential for inhibiting the protease enzymes that otherwise degrade barrier proteins like filaggrin.

The *Saccharomyces ferment*, however, offers a much broader metabolic spectrum, acting as a “biological factory” of micronutrients. It provides a dense cocktail of B-complex vitamins (B1, B2, B3, B5, B6, B12), essential amino acids, and minerals. Beyond basic nutrition, innovative research has identified yeast-derived metabolites (such as tryptophan derivatives) that act as ligands for the AhR. The AhR pathway is a master regulator of the skin barrier; its activation directly induces the expression of the FLG gene. Filaggrin is subsequently broken down into the amino acids that constitute the Natural Moisturizing Factor (NMF), thereby reducing TEWL. This trophic support ensures that the skin has the necessary biochemical precursors to rebuild itself from within [4,17,20,27].

Beyond basic nutrition, recent advances in postbiotic engineering have demonstrated the ability of specialized ferments to modulate the AhR pathway. This is a crucial mechanism for maintaining the skin barrier’s integrity. For instance, advanced exosomal-postbiotic complexes associated with plant extracts, such as the *Perilla*-derived exosomal complex (PEC), have shown the capacity to blunt CYP1A1 expression by up to 49% in human keratinocytes. This reduction in CYP1A1 activity is a key indicator of decreased oxidative stress and restored cellular homeostasis, which directly promotes the expression of the FLG gene. Consequently, this metabolic intervention addresses the fundamental biochemical deficiency in atopic dermatitis by enhancing the NMF and reducing TEWL [30,31].

### 3.4. Differential Modulation of the Skin Barrier: Antimicrobial Defense vs. Trophic Support

A critical aspect of postbiotic pharmacology in AD is the dual requirement for antimicrobial defense and structural repair. *Lactobacillus* postbiotics excel in pathogen exclusion. By utilizing Surface-layer (S-layer) proteins, these lysates anchor themselves to the epithelium, creating a physical and biochemical shield. This interaction induces “Quorum Quenching” mechanisms that disrupt the communication between *S. aureus* cells, significantly reducing biofilm formation and the secretion of pro-inflammatory exotoxins [32].

Simultaneously, *Saccharomyces ferments* provide trophic support that targets the underlying keratinocyte dysfunction. Peptide fractions and mannoproteins derived from yeast stimulate the proliferation of basal keratinocytes and the synthesis of collagen and loricrin. This accelerates the re-epithelialization of areas damaged by chronic scratching and inflammation. Furthermore, these fungal components exhibit potent antioxidant properties, ROS generated during inflammatory flares, thus protecting the skin’s DNA and lipid matrix from oxidative degradation [38].

The pharmacological distinction between the two kingdoms lies in their functional focus: while bacterial lysates are primarily oriented towards innate immune priming and the induction of AMPs, fungal ferments provide critical trophic support for the extracellular matrix. Quantitative assays on dermal fibroblasts have shown that these lysates can stimulate a 61.4% increase in Type I collagen synthesis and a remarkable 87.9% augmentation in elastin secretion. These findings, supported by Sirius Red and Elastin ELISA (Enzyme-Linked Immunosorbent Assay) reports, highlight the regenerative potential of yeast-derived postbiotics in repairing the damaged scaffolding matrix of atopic skin, offering an enhanced performance to tolerance ratio compared to traditional desquamating agents [36,39,40].

### 3.5. Synergistic Potential in Atopic Dermatitis: Rationale for Combined Therapy

The multifactorial nature of AD characterized by dysbiosis, barrier defect, and immune dysregulation—necessitates a multi-target therapeutic approach. The combination of bacterial and fungal lysates addresses these complexities more effectively than monotherapy. From a pharmacodynamic perspective, this synergy is manifested through the simultaneous modulation of the JAK–STAT and TLR signaling pathways [12]. The anti-inflammatory effect is evidenced by a 40% reduction in Interleukin-6 (IL-6) levels and a downregulation of CYP1A1 expression by up to 49% [30,31], effectively mitigating the chronic sub-clinical inflammation characteristic of AD [12,41,42].

While *Lactobacillus* fractions stabilize the innate immune response and suppress *S. aureus* overgrowth, *Saccharomyces* components stabilize the epidermal structure and provide the nutritional energy required for cellular repair. This “Multi-Kingdom” strategy restores the skin’s eubiosis and homeostasis, offering a sustainable alternative to long-term corticosteroid use [15,18,33,34,35].

The synergy of a “multi-kingdom” approach is validated not only by biochemical assays but also by in vivo clinical data. The anti-inflammatory effect is evidenced by a 40% reduction in IL-6 levels, effectively mitigating the chronic sub-clinical inflammation characteristic of AD. Furthermore, clinical evaluation using VISIA analysis has confirmed a measurable reduction in Red Areas (erythema) and a significant improvement in overall skin texture. This dual-action strategy—combining bacterial immune-modulation with fungal/exosomal structural repair—represents a robust pharmacological framework for the long-term management of atopic dermatitis. By targeting both the external microbiome and the internal cellular machinery, this combined postbiotic approach represents the next generation of precision skincare in dermatological therapy [41,42].

In conclusion, the pharmacological synergy between bacterial and fungal postbiotics provides a dual-action strategy that addresses both the microbial dysbiosis and the structural deficiencies inherent in atopic dermatitis. However, to fully understand the clinical efficacy of this multi-kingdom approach, it is essential to examine the underlying intracellular signaling pathways. The following chapter will explore the specific molecular mechanisms, with a particular focus on the modulation of the JAK–STAT and TLR pathways, which serve as the primary drivers of the cutaneous inflammatory cascade.

## 4. Molecular and Pharmacological Mechanisms of Action

### 4.1. Microbial Interference: Quorum Quenching and Biofilm Disruption

The cutaneous microbiome is an organized ecosystem where dominant microorganisms like *S. epidermidis* and *Cutibacterium acnes* maintain skin balance and immune homeostasis. In AD, this equilibrium is disrupted by a significant overcolonization of *S. aureus*, which contributes to disease exacerbation by breaching the epidermal barrier and inducing chronic inflammation. Factors such as elevated stratum corneum pH, filaggrin mutations, and decreased production of AMPs, like cathelicidins and beta-defensins, create a permissive environment for *S. aureus* adhesion and proliferation [44,45].

The pathogenicity of *S. aureus* is fundamentally governed by a density-dependent signaling mechanism known as Quorum Sensing (QS). At the core of this process lies the accessory gene regulator (Agr) locus, which coordinates the transition from commensal colonization to a high-virulence phenotype through the secretion of Autoinducing Peptides (AIPs). When the bacterial population reaches a critical threshold, the activation of the AgrC receptor triggers a feed-forward loop that drives the production of major virulence factors, including phenol-soluble modulin (PSMα) peptides and delta-toxin. These mediators induce the release of alarmins (IL-1α, IL-36α) from keratinocytes and cause mast cell degranulation, driving Th2/IL-17-mediated dermatitis [11,46,47].

In this context, PEC acts as a biomimetic postbiotic agent that disrupts *S. aureus* pathogenicity through a targeted Quorum Quenching (QQ) mechanism. This effect is primarily mediated by *Lactobacillus*-derived exosomes—bacterial extracellular vesicles (30–150 nm) that function as specialized nano-delivery vehicles [48]. These exosomes encapsulate bioactive peptides and metabolites that act as competitive antagonists by binding to the AgrC receptor, effectively “jamming” the signal transduction required for quorum sensing.

By utilizing this vesicle-mediated delivery, PEC effectively silences staphylococcal communication and reduces the toxin load without the selective pressure associated with traditional bactericidal agents [49]. This inhibition prevents the bacteria from activating the Agr system, thereby halting the transition to a virulent state and the subsequent formation of the protective biofilm matrix. Furthermore, these exosomal cargos interfere with the ica operon, reducing the production of polysaccharide intercellular adhesin (PIA) essential for biofilm integrity [50,51,52]. Consequently, this “silencing” strategy restores the competitive equilibrium of the skin microbiome and protects the epidermal structure from protease-mediated dissolution, offering a safe and stable therapeutic alternative for AD management.

### 4.2. Innate Immunity Training: TLR-2 Modulation and Antimicrobial Peptide Induction

Keratinocytes provide structural integrity and act as immune-competent cells that actively monitor and respond to microbial invasions. A key mechanism of this defense is the synthesis and storage of AMPs, which eliminate diverse pathogens while simultaneously strengthening and improving the skin barrier integrity [53]. The most significant AMPs involved in these pathways include cathelicidins (LL-37), beta-defensins (hBD-2 and hBD-3), dermicidin, and CXCL14—a unique chemokine that binds bacterial DNA, such as that of *S. aureus* [54].

Keratinocytes detect pathogens via pattern recognition receptors (PRRs), primarily Toll-like receptors (TLRs). TLRs recognize pathogen-associated molecular patterns (PAMPs) like lipids, lipoproteins, and nucleic acids [55]. In AD, an imbalance in AMP expression (lower LL-37, hBD-2, and dermicidin-1 levels) promotes dysbiosis and microbial colonization [56,57]. This deficiency is partly attributed to the overexpression of Th2 cytokines (IL-4, IL-13, IL-31), which inhibit AMP production and suppress filaggrin [57].

In this context, the development of postbiotic interventions like PEC represents a biomimetic strategy for “innate immunity training.” PEC utilizes a synergistic exosomal complex comprising *Lactobacillus*-derived fractions and *Perilla frutescens* extract, encapsulated within a phospholipid nanovesicular system [48].

Unlike simple lysates, this exosomal delivery system optimizes the interaction with TLR2, a key receptor for skin barrier repair.

Research on *Lactobacillus*-derived postbiotics indicates that bioactive substances like LTA have important immunomodulatory properties when interacting with TLR2. In the case of PEC, the exosomal encapsulation ensures that these *Lactobacillus*-derived ligands act as partial agonists, “priming” keratinocytes to reinforce innate defenses without triggering the hyper-inflammation typically induced by pathogens like *S. aureus* [54,57]. The complex interacts with the TLR2/6 heterodimer, initiating signaling pathways that upregulate essential tight junction proteins, including ZO-1, Occludin, and Claudin-1, effectively repairing the “leaky” epidermal barrier [8,58,59].

Furthermore, the presence of *Perilla frutescens* phytochemicals within the PEC provides a potent anti-inflammatory synergy. By binding to TLR2, the postbiotic ligands effectively outcompete and block the signaling cascade normally triggered by pathogenic *S. aureus* LTA, a critical mechanism for alleviating atopic symptoms. This competitive inhibition prevents the overproduction of CCL2 (Chemokine ligand 2) and reduces the activation of the ERK and p38 MAPK pathways, leading to a significant mitigation of the pro-inflammatory environment. Specifically, experimental assays using ELISA techniques demonstrated a 40% reduction in Interleukin-6 (IL-6) levels [4], alongside decreased levels of TNF α, IL-8, IL-4, and IL-5, and an enhanced expression of the anti-inflammatory mediator IL-10 [60].

Beyond barrier repair, PEC promotes microbial homeostasis by selectively inhibiting the overgrowth of pathogenic *S. aureus* while supporting beneficial commensals. Clinical applications of these stable, exosome-encapsulated *Lactobacillus* fractions demonstrate significant texture improvement and reduction in red areas in atopic skin models [42], offering a safe therapeutic alternative to live probiotics and demonstrating significant reductions in the SCORAD index and TEWL [61].

### 4.3. Targeting the JAK–STAT Pathway: Biomimetic Delivery via Perilla frutescens-Derived Exosomes

The therapeutic efficacy of the studied complex is anchored in a multi-target biomimetic approach, specifically designed to modulate the intracellular signaling cascades that orchestrate the pathogenesis of AD. By utilizing exosomal vesicles enriched with *Perilla frutescens* bioactives, the formulation achieves superior precision in targeting the JAK–STAT axis and the regulatory checkpoints of the skin’s immune response.

#### 4.3.1. Modulation of the JAK/STAT Signaling Axis

The JAK/STAT (Janus Kinase/Signal Transducer and Activator of Transcription) pathway is the primary transducer for a broad range of cytokines involved in AD. The exosomal delivery of concentrated bioactives targets two major intracellular signaling axes:

1. The Th2 Axis (Acute Phase): IL-4 and IL-13 signal through JAK1/JAK3 and JAK1/TYK2 to activate STAT6, driving IgE production and inflammatory cell recruitment [11,12].
2. The Th22/Th17 Axis (Chronic Phase): IL-22 and IL-23 utilize JAK1, JAK2, and TYK2 to activate STAT3, driving pathological epidermal thickening (acanthosis) and tissue remodeling [12,46].

This molecular regulation is clinically supported by the acceleration of cellular renewal [58], demonstrating that blocking STAT3-mediated hyperproliferation allows for a controlled, physiological regeneration of the skin barrier.

#### 4.3.2. The SOCS Regulatory Framework: Biological Checkpoints

The SOCS (Suppressors of Cytokine Signaling) protein family, particularly SOCS1-7 and CIS, serves as a critical regulatory mechanism for managing cytokine-driven inflammation. These molecules act as natural “brakes,” preventing overactive JAK kinase activation. In the context of AD, SOCS1, SOCS3, and SOCS5 act as essential checkpoints that modulate the balance between Th1 and Th2 cells, effectively attenuating Th2-mediated allergic inflammation [12]. The postbiotic complex stabilizes this regulatory framework, preventing the hyperactive immune responses that lead to chronic lesions.

#### 4.3.3. Synergistic Action of Perilla-Derived Bioactives: Luteolin and Rosmarinic Acid

The biomimetic delivery of *Perilla frutescens* bioactives via exosomes ensures that they bypass the stratum corneum to interact directly with keratinocyte and immune cell receptors (see Table 2):

- Luteolin (Antipruritic and Immunomodulator): Acts as a potent inhibitor of STAT3 and modulates the release of TSLP (Thymic Stromal Lymphopoietin). By blocking TSLP signaling, luteolin breaks the “itch-scratch cycle” and reduces mechanical-induced inflammation [62,63]. This action is validated by a report [49] confirming the preservation of total cellular protein integrity under environmental stress.

Rosmarinic Acid (Barrier Restoration and Antioxidant) mechanisms:

- Inhibition of IKK-β Signaling: Rosmarinic acid exerts a targeted immunomodulatory effect by suppressing the production of key chemokines, such as CCL11 (eotaxin), and its corresponding receptor CCR3. This molecular blockade effectively limits eosinophil infiltration into the dermal layers, thereby attenuating the acute allergic response characteristic of atopic flares [12,13].
- Activation of the Nrf2/HO-1 Axis: This compound fortifies the skin’s internal resilience by upregulating endogenous antioxidant defenses within keratinocytes. By activating the Nrf2/HO-1 pathway, it neutralizes ROS and significantly reduces the cellular damage induced by oxidative stress [64].
- Stimulation of NHE1 and pH Regulation: A pivotal role of Rosmarinic acid in physical barrier reconstruction involves the activation of the Sodium-Proton Exchanger 1 (NHE1). This stimulation decreases skin surface pH to restore the “acid mantle” and increases ceramide levels, providing the necessary biochemical environment for the observed upregulation of collagen [40] and elastin [39] synthesis [44,65].
- Advanced Anti-inflammatory Pathways: Rosmarinic acid further modulates systemic inflammation by suppressing the production of TACE (TNF-alpha-converting enzyme), which prevents the shedding of EPCR (Endothelial Protein C Receptor) and the subsequently regulated release of TNF-alpha. Additionally, its capacity to interfere with the HMGB1 (High Mobility Group Box Protein 1) signaling pathway underscores its potential in managing severe and recalcitrant inflammatory disorders [66,67].

**Table 2:** Key Molecular Targets and Signaling Pathways Modulated by *Perilla frutescens*-derived Bioactive Compounds.

Functional Axis	Bioactive Compound	Molecular Target/Pathway	Mechanism of Action	Biological Effect in Atopic Dermatitis	References
Immune Modulation	Luteolin	STAT3 signaling	Inhibits STAT3 activation involved in inflammatory signaling	Reduces inflammatory cytokine signaling and immune activation	[62,63]
Immune Modulation	Luteolin	TSLP	Blocks TSLP signaling in keratinocytes	Interrupts the itch–scratch cycle and decreases pruritus-induced inflammation	[62,63]
Immune Modulation	Rosmarinic Acid	IKK-β/NF-κB pathway	Inhibits IKK-β activity and downstream inflammatory signaling	Reduces chemokines such as CCL11 (eotaxin) and limits CCR3-mediated eosinophil recruitment	[12,13]
Immune Modulation	Rosmarinic Acid	TACE	Suppresses TNF-α activation and prevents EPCR shedding	Reduces inflammatory cytokine release and vascular inflammation	[66,67]
Immune Modulation	Rosmarinic Acid	HMGB1 signaling	Interferes with HMGB1 inflammatory signaling cascade	Limits severe inflammatory responses and tissue damage	[66,67]
Antioxidant Defense	Rosmarinic Acid	Nrf2/HO-1 axis	Activates transcription of antioxidant enzymes	Neutralizes ROS and reduces oxidative stress in keratinocytes	[64]
Barrier Restoration	Rosmarinic Acid	NHE1	Stimulates NHE1 activity regulating epidermal pH	Lowers skin surface pH and increases ceramide production	[51,65]
Barrier Restoration	Rosmarinic Acid	Extracellular matrix synthesis	Promotes structural protein production	Upregulates collagen and elastin, improving dermal integrity	[39,40]
Cellular Protection	Luteolin	Cellular protein stability pathways	Preserves total cellular protein integrity under environmental stress	Protects keratinocytes from stress-induced damage	[49]

**Abbreviations**: TSLP (Thymic Stromal Lymphopoietin), TACE (TNF-α-converting enzyme), NHE1 (Na^+^/H^+^ Exchanger 1).

#### 4.3.4. Targeted Biomimetic Delivery and Clinical Outcomes

The efficacy of these thirteen distinct molecular pathways is intrinsically linked to the exosomal delivery system. By encapsulating these bioactives within nanovesicles (30–150 nm), the formulation ensures protection against proteolytic degradation and facilitates targeted delivery to deep epidermal layers. The final synergistic result is a measurable restoration of skin texture and the lipid mantle, as confirmed by VISIA analysis [42], offering a high-tech alternative to the simple occlusion provided by traditional emollients [41,59].

### 4.4. Epidermal Barrier Restoration: PPAR-γ Activation and Lipid Homeostasis

The modern management of AD necessitates a proactive restoration of the skin barrier, a process orchestrated by the bioactive constituents of *Perilla frutescens*—Alpha-Linolenic Acid (ALA), Rosmarinic Acid, and Luteolin—which function as potent bio-activators of lipid homeostasis.

#### 4.4.1. PPAR-γ Signaling and Lipid Synthesis

Alpha-Linolenic Acid (ALA), extracted from *Perilla* seeds, acts as a direct ligand for PPAR-γ (Peroxisome Proliferator-Activated Receptor gamma) within keratinocytes. This activation triggers the genetic signaling required for keratinocyte maturation, ensuring an organized transition from the basal layer to the stratum corneum [68,69]. ALA promotes the synthesis of endogenous ceramides and essential fatty acids, fortifying the intercellular matrix. This biochemical fortification significantly reduces TEWL, a result clinically validated by VISIA analysis [70], which demonstrates a measurable restoration of the lipid mantle and skin micro-relief.

Rosmarinic Acid complements this process by serving as a critical stabilizer that prevents the peroxidation of surface lipids. By activating the Nrf2 signaling pathway, it protects filaggrin from enzymatic degradation and maintains lipid fluidity, preventing the formation of micro-fissures [38,53]. This protection of structural protein integrity is further supported by total cellular protein assays [50] confirming the resilience of the proteome under oxidative stress.

#### 4.4.2. Structural Integrity and Cellular Renewal

Flavonoids such as Luteolin and Apigenin accelerate keratinocyte differentiation by stimulating the expression of loricrin and involucrin. This structural reinforcement is evidenced by an increased rate of cellular renewal [2], providing the mechanical resilience necessary for fragile AD skin. Furthermore, the synergy between PPAR-γ activation and NF-κB inhibition reduces the production of pro-inflammatory cytokines, stabilizing the epidermal metabolic environment.

#### 4.4.3. Exosomal Delivery and ECM Remodeling

The use of plant-derived nanovesicles as biomimetic delivery systems (30–150 nm) facilitates the deep transport of these compounds, protecting them from proteolytic degradation. Beyond immune modulation, these nanovesicles actively stimulate the synthesis of collagen and elastin while inhibiting matrix metalloproteinases (MMPs) [39,40,64].

**Figure 1:**
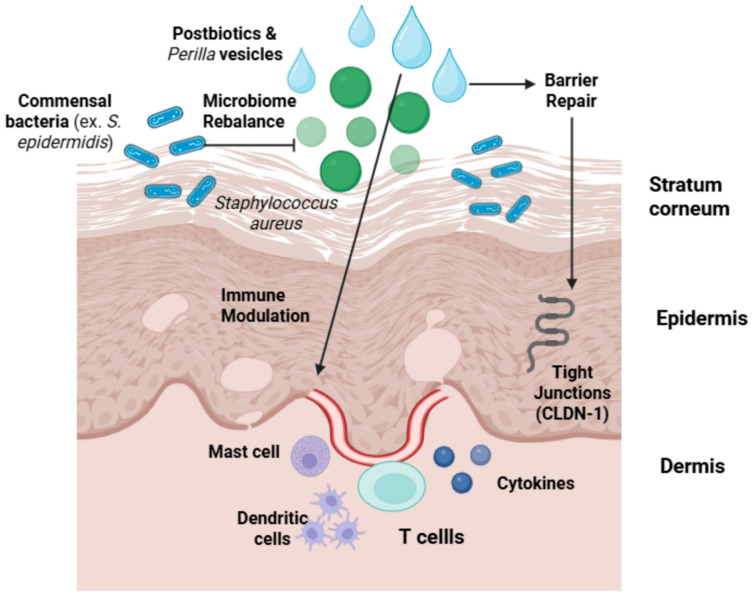
Mechanisms of the multi-kingdom synergy (postbiotics and *Perilla frutescens*-derived vesicles) in AD. The complex targets three primary pathological levels: (1) Microbiome Rebalance, by inhibiting *S. aureus* (in green) and supporting commensal flora (e.g., *S. epidermidis*, in blue); (2) Barrier Repair, by reinforcing stratum corneum integrity and upregulating Tight Junctions (CLDN-1); and (3) Immune Modulation, by suppressing inflammatory signaling in the Dermis. Created by the authors with BioRender.com. **Abbreviations**: AD, Atopic Dermatitis, CLDN-1, Claudin-1, *S. epidermidis*, *Staphylococcus epidermidis*.

As illustrated in Figure 1, the therapeutic strategy of the PEC is based on a multi-targeted approach designed to restore skin homeostasis in AD. This multi-kingdom synergy operates at three distinct pathological levels:

1. Microbiome Rebalance: The complex effectively inhibits the overproliferation of *S. aureus* while simultaneously supporting the growth of beneficial commensal flora, such as *S. epidermidis* and *S. hominis*, thereby restoring microbial diversity [2,14].
2. Barrier Repair: By upregulating key structural proteins such as Claudin-1 (CLDN-1), the complex reinforces the tight junctions and the integrity of the stratum corneum, reducing TEWL [8,58].
3. Immune Modulation: The bioactive cargo delivered by the exosomal system acts within the dermis to suppress pro-inflammatory signaling via the JAK–STAT pathway, modulating the activity of mast cells and T-cells to limit cytokine-induced inflammation [11,12,62].

This comprehensive approach ensures that the skin is not only protected from dysbiosis but is also structurally and nutritionally supported for long-term barrier recovery [52].

## 5. Comparative Analysis: Postbiotics vs. Conventional Therapies in AD Management

The current therapeutic landscape for AD is characterized by a high reliance on reactive treatments. However, as the therapeutic pipeline expands [71], there is a growing need for proactive strategies that address the microbial and immunological roots of the disease. This chapter evaluates exosomal postbiotics against established standards—antibiotics and corticosteroids—and highlights their role in modern precision medicine.

### 5.1. Precision Ecology: Postbiotics vs. Antibiotics and Antiseptics

The management of *S. aureus* overcolonization is a cornerstone of AD therapy, yet global susceptibility patterns indicate an alarming increase in multi-drug-resistant strains [72]. Conventional topical antibiotics exert selective pressure, further driving this resistance and inducing profound dysbiosis by eliminating beneficial commensals. Furthermore, the use of non-selective antiseptics can disrupt the delicate signaling required for skin homeostasis [46].

PEC represents a shift toward “Precision Ecology.” By utilizing exosomal cargo to interfere with the *agr*-mediated Quorum Sensing of *S. aureus* [49], it effectively “disarms” the pathogen without bactericidal pressure (see Table 3). This prevents the formation of virulent biofilms and the secretion of dermonecrotic toxins [46]. Unlike antibiotics, this postbiotic shield preserves the commensal landscape, allowing the host’s microbiome to maintain its protective niche and prevent pathogen re-colonization [72].

While *Lactobacillus* fractions focus on neutralizing *S. aureus* virulence, the *Saccharomyces ferment* component within the PEC provides a unique ecological advantage. By secreting specific enzymes and antioxidant metabolites, it creates a nutrient-rich environment that favors the regrowth of beneficial commensals like *S. epidermidis*, effectively outcompeting pathogens through resource competition rather than chemical toxicity.

### 5.2. The “Steroid-Sparing” Potential: Postbiotics as Adjuvant and Proactive Therapy

While topical corticosteroids (TCS) are effective for acute flares, “steroid phobia” remains a significant barrier to long-term management, leading to poor adherence and chronic disease cycling [73]. The development of “steroid-sparing” agents is therefore a clinical priority [34].

The integration of *Perilla frutescens*-derived bioactives within the PEC offers a potent anti-inflammatory alternative. Luteolin, the primary flavonoid in this complex, has been shown to inhibit the JAK–STAT pathway, specifically suppressing IL-6 and Th2-driven inflammation [74]. This molecular modulation mimics the efficacy of mild corticosteroids but without the risks of skin atrophy or rebound effects (see Table 3). Used as a proactive therapy, this complex stabilizes the immune response, significantly prolonging flare-free intervals and reducing the cumulative need for TCS [73,74,75].

### 5.3. Beyond pH Correction: Complex Postbiotic Cocktails vs. Simple Acidification

Restoring the skin’s “acid mantle” is a traditional goal in AD care, often achieved through simple acidification with lactic or citric acid. However, surface pH correction alone is insufficient to repair the deep structural and signaling defects of the AD barrier [17].

The ‘Postbiotic Cocktail’ approach in PEC delivers a multi-dimensional restorative signal. Unlike simple acidic emollients, the *Saccharomyces*-derived β-glucans actively stimulate the Dectin-1 receptors on keratinocytes, triggering the endogenous synthesis of filaggrin and loricrin [26,38]. This bio-inductive process addresses the underlying structural ‘leakiness’ of the AD barrier. Furthermore, the fungal ferment acts as a metabolic primer, enhancing cellular ATP levels to provide the energy required for the upregulated synthesis of collagen and elastin [18,19,39,40], ensuring a biological repair that goes beyond mere surface coating [27], and transforming the skin from a state of chronic inflammation to one of active structural remodeling [11,42].

### 5.4. Safety Profile and Long-Term Compliance: Overcoming Traditional Limitations

Adherence to topical regimens is notoriously hindered by the poor sensory properties of traditional emollients (e.g., greasiness, stickiness) and the potential for local irritation [76]. For many patients, the “medical” feel of traditional ointments acts as a deterrent to daily use.

Exosomal postbiotics overcome these compliance barriers through advanced formulation technology [7]. PEC is validated for high cellular biocompatibility [77], making it suitable for even the most compromised skin types. The nanovesicular structure ensures rapid, deep absorption with a superior cosmetic finish, which significantly enhances patient adherence [76]. This is further supported by VISIA analysis [42], which demonstrates a measurable improvement in skin microrelief and lipid mantle restoration, providing the patient with both clinical relief and a visible aesthetic benefit.

While these results confirm the potency of the bacterial exosomal cargo, the potential integration of *Saccharomyces ferment* in advanced therapeutic protocols offers a further synergistic advantage. By providing a “metabolic boost” through ATP production and stimulating the synthesis of structural proteins like filaggrin, fungal components could theoretically augment the visible clinical benefits observed with PEC alone, leading to an even more robust and long-lasting barrier repair.

Despite these clinical and sensory advantages, several translational limitations must be acknowledged. First, while the high biocompatibility of PEC is validated [77], long-term studies are necessary to ensure that continuous modulation of the skin microbiome does not lead to unintended shifts in commensal diversity. Furthermore, from a safety perspective, the use of nanovesicular structures in compromised skin (typical of AD) requires rigorous monitoring to exclude any potential for systemic absorption of the exosomal cargo.

Finally, regulatory challenges remain a significant hurdle for multi-kingdom postbiotic therapies. The classification of plant-derived vesicles and bacterial ferments often falls between cosmetic and pharmaceutical frameworks, necessitating standardized manufacturing (GMP) and precise batch-to-batch biochemical characterization to meet international safety standards.

## 6. Challenges in Pharmaceutical Technology and Formulation Stability

The transition from theoretical pharmacology to clinical efficacy in AD depends critically on the stability of bioactives and their ability to penetrate the stratum corneum. As the therapeutic pipeline for AD expands [72], technological challenges focus on developing delivery systems that protect fragile molecules against degradation while maintaining the skin’s ecological balance.

### 6.1. Incorporating Postbiotics into O/W Emulsions: Preserving Protein Bioactivity

Incorporating postbiotics into Oil-in-Water (O/W) emulsions presents a significant challenge: maintaining the structural integrity of bacterial metabolites, enzymes, and peptides. Conventional emulsification processes often expose these thermolabile compounds to mechanical and thermal stress, leading to a rapid loss of bioactivity [7].

In the formulation of the studied complex, this challenge is addressed through exosomal encapsulation. These bacterial extracellular vesicles (EVs) function as specialized nano-delivery vehicles that encapsulate bioactive peptides and autoinducer-like molecules, protecting them from proteolytic degradation within the skin’s complex enzymatic environment [49,50]. According to molecular assays, this stabilization ensures the preservation of the total cellular protein profile [50]. By shielding these “messenger” molecules, the system guarantees that the postbiotic cargo remains functional until it reaches the targeted epidermal layers, effectively bridging the gap between raw fermentation products and stable cosmetic vehicles.

In addition to bacterial metabolites, the incorporation of *Saccharomyces ferment* introduces a complex matrix of β-glucans and essential vitamins (B-complex) into the O/W emulsion [43,44]. While bacterial peptides are highly thermolabile, these fungal polysaccharides exhibit superior structural stability under mechanical stress [25,26]. When co-formulated within the exosomal complex, the *Saccharomyces* fractions act as a secondary stabilizing network, physically protecting the more fragile exosomal vesicles from premature coalescence and ensuring the preservation of the holistic ‘Postbiotic Cocktail’ bioactivity [49,78].

### 6.2. The Formulation Paradox: Developing Microbiome-Friendly Products

A major paradox in modern pharmaceutical technology is the use of broad-spectrum preservatives, which, while ensuring the microbiological stability of the formulation, may inadvertently disrupt the host’s commensal microbiota [79]. Developing microbiome-friendly products requires a delicate balance between product safety and prebiotic/postbiotic support [7].

PEC addresses this paradox by utilizing metabolites that inherently support a healthy cutaneous environment. Biosurfactants and exopolysaccharides derived from *Lactobacillus ferment* create an anti-adhesive layer that prevents the integration of the pathogen *S. aureus* into biofilms [49,51]. Furthermore, the endogenous production of lactic acid lowers the skin pH to physiological levels, subsequently disabling the acid-sensitive agr signaling system of the staphylococcus [48]. By utilizing these postbiotic stabilizers, the formulation supports the growth of beneficial species, promoting long-term immune homeostasis rather than temporary bactericidal effects that can trigger resistance [79].

The inclusion of *Saccharomyces ferment* further bolsters this ‘microbiome-friendly’ profile. Unlike synthetic preservatives that act indiscriminately, the fermentation by-products of *Saccharomyces*—specifically organic acids and amino acids—exert a natural self-preserving effect on the formulation while simultaneously acting as prebiotics [7,38]. This selectively supports the growth of *S. epidermidis* over *S. aureus*, reinforcing the skin’s natural ‘acid mantle’ without the need for high-dose traditional biocides that could trigger irritation in AD patients [79,80].

### 6.3. Exosomal Stability and Controlled Release: Ensuring Targeted Delivery

**Figure 2:**
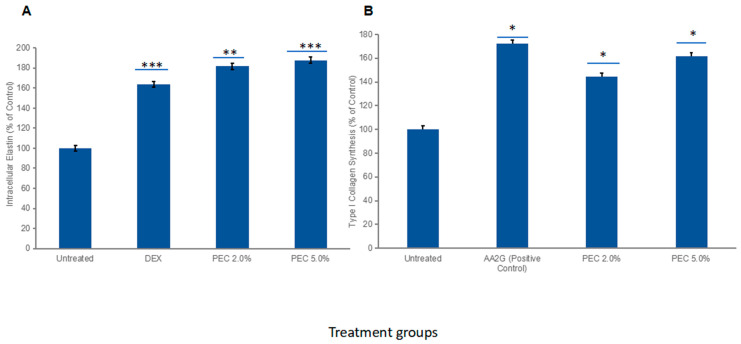
Evaluation of the PEC on extracellular matrix synthesis. (**A**) Intracellular elastin synthesis in dermal fibroblasts; (**B**) Type I collagen synthesis measured via ELISA. AA2G and DEX were used as positive controls for collagen and elastin, respectively. Results are expressed as mean ± SD (*n* = 3). Statistical significance was determined using one-way ANOVA (* *p* < 0.05, ** *p* < 0.01, *** *p* < 0.001 vs. untreated control). Data sourced from manufacturer technical validation studies [39,40]. **Abbreviations**: PEC, Postbiotic and Exosomal Complex; DEX, Dexamethasone; AA2G, Ascorbic Acid 2-Glucoside; ELISA, Enzyme-Linked Immunosorbent Assay; SD, Standard Deviation; ANOVA, Analysis of Variance.

Nanotechnology-based topical delivery systems have substantially improved skincare by overcoming the skin’s natural barrier function [81]. Exosomes, with controlled nanometric dimensions (30–150 nm), represent the “gold standard” of biomimetic delivery, facilitating intercellular communication through the direct transfer of proteins and nucleic acids to target cells [54,64]. The stability of these nanovesicles in PEC allows for a controlled and sustained release of the bioactive cargo. Unlike free lysates that remain on the skin surface, the encapsulated ligand ensures deep penetration and sustained interaction with immune receptors. A crucial mechanism identified is the ability of exosomes to induce the transition of macrophages from the pro-inflammatory M1 phenotype to the reparative M2 phenotype, thereby reducing chronic inflammation [64,82]. The therapeutic potential of the PEC in modulating the skin’s structural architecture was validated through the significant upregulation of extracellular matrix (ECM) components. As illustrated in Figure 2, the complex induced a robust, dose-dependent increase in both elastin and collagen synthesis. Specifically, treatment with 5.0% PEC resulted in an 87.9% net increase in intracellular elastin (Figure 2A), reaching a total relative expression of 187.9% compared to the untreated control (*p* < 0.001). This effect outperformed the positive control (Dexamethasone), which showed a 63.7% increase. Similarly, Figure 2B demonstrates that the PEC significantly enhanced Type I collagen synthesis, with the 5.0% concentration achieving a 61.4% net increase (totaling 161.4% of control, *p* < 0.05), a performance comparable to the gold-standard antioxidant L-ascorbic acid (72.5% increase) [39,40].

These findings suggest that the delivery efficiency of the exosomal system, combined with the metabolic priming effect of the *Saccharomyces* ferment, successfully accelerates cellular renewal and reinforces the skin’s structural integrity [39,40,42,59].

By providing the necessary cellular energy (ATP) required for protein synthesis, the fungal component ensures that once the exosomes deliver their signaling cargo (lipids and peptides) to the fibroblasts, the cells have the energetic substrate to execute the ‘repair command’ [19,27].

The clinical efficacy of the PEC is fundamentally rooted in its superior stability and targeted delivery system.

**Figure 3:**
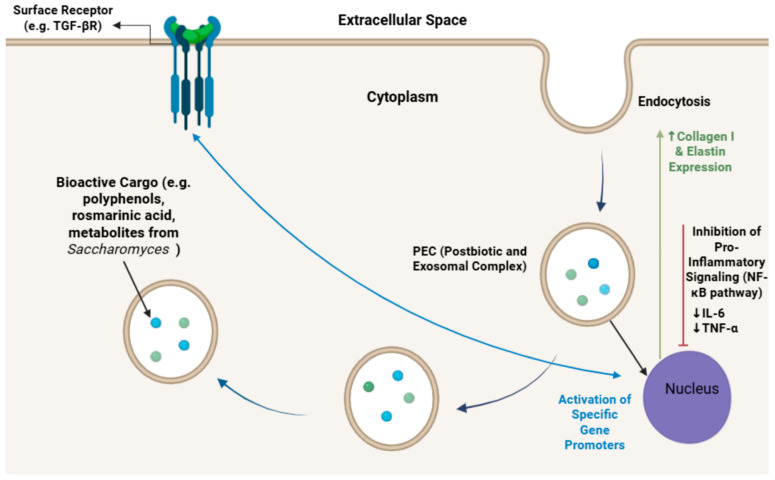
Comprehensive molecular mechanism of the PEC in human dermal fibroblasts. The complex employs a synergistic delivery and signaling strategy: (1) internalisation of bioactive cargo (polyphenols, rosmarinic acid, and yeast metabolites) via endocytosis, and (2) direct interaction with surface receptors (e.g., TGF-βR). These pathways converge to inhibit pro-inflammatory NF-κB signaling (downregulating IL-6 and TNF-α) and activate specific gene promoters in the nucleus, ultimately leading to the robust upregulation of Type I Collagen and Elastin expression. Created by the authors with BioRender.com. **Abbreviations**: PEC, Postbiotic and Exosomal Complex; TGF-βR, Transforming Growth Factor-beta Receptor; NF-κB, Nuclear Factor kappa-light-chain-enhancer of activated B cells; IL-6, Interleukin-6; TNF-α, Tumor Necrosis Factor-alpha.

As illustrated in Figure 3, the PEC utilizes its exosomal nanostructure (30–150 nm) as a biomimetic carrier, preventing the premature degradation of sensitive polyphenols and postbiotic metabolites. This ensures a controlled delivery across the epidermal layers, allowing the bioactive compounds to reach the deeper dermis.

Beyond acting as a protective carrier, the complex exerts a dual molecular effect upon reaching the dermal fibroblasts: it facilitates the entry of bioactive cargo via endocytosis and simultaneously interacts with surface receptors (e.g., TGF-βR). This synergistic activation triggers an intracellular signaling cascade that inhibits pro-inflammatory pathways (downregulating IL-6 and TNF-α) while stimulating the nuclear expression of Type I Collagen and Elastin, thereby driving the observed remodeling effects on the extracellular matrix.

These results collectively demonstrate the potent regenerative capacity of the PEC. Furthermore, the clinical relevance of these findings is supported by the safety profile of the ingredients; cytotoxicity assays performed by the manufacturer confirmed that the PEC is safe for topical applications, showing no adverse effects on cell viability at the tested concentrations (2.0–5.0%). This balance between high biological activity and cellular safety makes PEC a promising candidate for long-term treatment of inflammatory skin conditions.

## 7. Future Perspectives: Toward Precision Postbiotics in Dermatology

### 7.1. Beyond “Lysates”: The Need for Standardization via Proteomics and Metabolomics

Postbiotics are commonly referred to by general terms (such as “lysates”), but this nomenclature can mask significant variation in the bioactive makeup of different products and batches. By defining repeatable molecular “fingerprints”, omics-based characterization (proteomics and metabolomics, supplemented by multi-omics approaches) promotes quality control, cross-study comparability, and more reliable mechanistic interpretation. Such standardization becomes crucial for connecting particular postbiotic components to clinically significant outcomes, such as barrier recovery and modulation of *S. aureus*–associated dysbiosis in atopic dermatitis, where host-microbiome interactions and immune responses vary over time [19].

In atopic dermatitis, characterized by dynamic host-microbiome interactions and immune responses, multi-omics facilitates the correlation of microbial and metabolic signals with clinical biomarkers (severity, endo/phenotypes, therapy response). In this context, omics-based standardization is not merely an analytical endeavor but a necessity for comparing postbiotic interventions and identifying components linked to significant effects (anti-inflammatory, barrier restoration, modulation of *S. aureus* colonization) [20,83].

Upon standardization via omics, postbiotics can be more effectively incorporated into precision medicine frameworks, wherein patient profiling and endophenotype stratification gain significance [83,84].

### 7.2. Personalized Postbiotics: Microbiome Profiling and Tailored Therapeutic Interventions

AD demonstrates significant clinical and biological variability, advocating for a transition from uniform treatment approaches to tailored, profile-informed adjunctive strategies. Profiling the skin microbiome has consistently associated atopic dermatitis activity with dysbiosis patterns, typically marked by diminished microbial diversity and alterations in microbial composition that correlate with barrier dysfunction and inflammation, underscoring the microbiome as a clinically significant aspect for disease characterization and monitoring [85].

In this context, postbiotics may serve as profile-guided adjuncts intended to restore a healthier skin ecosystem and facilitate barrier recovery. Postbiotics-comprising specific bioactive constituents from microbial origins–are progressively recognized as instruments to influence host-microbe interactions, mitigate dysbiosis-related inflammatory signaling, and enhance conventional management of atopic dermatitis. Thus, personalization can be effectively implemented by selecting and adjusting postbiotic regimens according to patient-specific factors (flare propensity, barrier integrity, and microbiome characteristics), aiming to enhance stability and decrease relapse rates [86].

A clinically valuable profiling framework necessitates the integration of microbiome signals with recognized disease biomarkers and endotype/phenotype stratification, in addition to descriptive dysbiosis. The severity and progression of atopic dermatitis are influenced by barrier integrity, the intensity of type 2 inflammation, microbial colonization patterns, and individual patient factors, including flare propensity and previous treatment history. Thus, “customized therapeutic interventions” must be based on quantifiable metrics (clinical scores, barrier assessments, and biomarker panels), enabling clinicians to categorize postbiotics as adjunctive, proactive, or maintenance strategies instead of standardized, reactive approaches. This method facilitates the rational selection of postbiotic formulations and dosing regimens tailored to the predominant pathophysiological factors in each patient group, while allowing for continuous monitoring of responses and prompt modifications upon the emergence of deterioration indicators [84,87,88].

From a therapeutic perspective, personalization can be operationalized at three levels: (1) treatment timing (reactive use during flares vs. proactive, intermittent maintenance to prevent recurrence), (2) formulation choice (single active fractions vs. multi-component postbiotic blends. Depending on whether the goal is anti-inflammatory modulation, barrier repair, or microbial interference), and (3) target selection (microbiome rebalancing in *S. aureus*–dominant dysbiosis vs. barrier reinforcement in patients with marked xerosis and increased transepidermal water loss). Crucially, by matching postbiotic interventions with the patient’s microbial and barrier context and with useful endpoints like increased tolerability, adherence, and decreased relapse frequency, the personalized concept refines adjunctive care rather than replacing proven anti-inflammatory therapies [19,84,87,88,89].

To maintain clinical relevance, the utilization of profiling-informed postbiotics should be contextualized within guideline-based management and regarded as adjunctive care that enhances the fundamental treatment components for atopic dermatitis (emollients, anti-inflammatory agents, trigger management, and proactive approaches for appropriate patients). A principal benefit of a profiling-based methodology is the capacity to elucidate the rationale behind the selection of a specific postbiotic regimen, the criteria for measuring success (clinical severity, flare rate, barrier markers, and/or microbiome alterations), and the appropriate timing for escalation or de-escalation. In this context, tailored postbiotic protocols can be incorporated into organized care pathways, improving decision-making consistency while maintaining adaptability for individual patient requirements [84,85,88].

### 7.3. The Integration of AI in Predicting Postbiotics Efficacy

Artificial intelligence (AI) and machine learning can facilitate the precision-driven application of postbiotics by synthesizing diverse data that is challenging to analyze manually. In atopic dermatitis, predictive models may integrate clinical variables (baseline severity, flare history, treatment exposure), objective indicators of disease activity (standardized severity scoring), and longitudinal data concerning barrier integrity or microbial patterns to assess flare risks and likelihood of response. AI tools ought to be regarded as decision-support instruments that enhance—rather than supplant—clinical judgment, with their efficacy contingent upon transparent inputs, external validation, and clinically significant endpoints [90,91].

Current research shows useful paths related to this strategy. While machine learning-based deep phenotyping can uncover latent patient subgroups with unique trajectories, automated or AI-assisted severity assessment can lessen inter-observer variability and enable consistent monitoring over time. Simultaneously, passive flare-detection systems and digital biomarkers suggest that early warning indicators may be recorded over time and converted into proactive modifications of adjunctive measures, such as postbiotic regimens. The next step will be prospective workflows where AI-guided stratification is explicitly linked to predefined treatment pathways and patient-centered outcomes as standardized datasets (clinical, omics, and microbiome-linked features) grow [92,93].

Beyond standalone applications, the integration of postbiotics into hybrid therapeutic protocols represents a significant shift in AD management. While systemic biological agents effectively suppress the internal inflammatory cascade (an “inside-out” approach), the adjunctive use of exosomal postbiotics offers a synergistic “outside-in” restoration. By specifically targeting the AhR-mediated synthesis of filaggrin and promoting long-term barrier bio-induction, postbiotics may enhance the clinical longevity of biological treatments and facilitate a “steroid-sparing” strategy, potentially reducing the frequency of flares in chronic patients.

In this hybrid framework, topical exosomal postbiotics serve as the cornerstone of the ‘outside-in’ restorative strategy. Unlike traditional lysates, exosome-encapsulated ligands provide enhanced stability and deeper penetration into the stratum corneum, directly interacting with the AhR. This interaction bio-induces the endogenous synthesis of filaggrin and loricrin, effectively sealing the physical barrier from the exterior. By blunting oxidative stressors such as the CYP1A1 pathway, these exosomal systems neutralize environmental triggers before they can activate the internal immune cascade, thus providing a proactive shield that complements systemic modulation. This proactive shield is further enhanced by the inclusion of *Saccharomyces*-derived postbiotics, which address the metabolic exhaustion of atopic keratinocytes. While the bacterial exosomal cargo in PEC modulates the immune response [33,61], the fungal ferment provides the essential energetic substrate (ATP) required for the bio-induction of structural proteins [27,43]. Specifically, yeast-derived peptides and mannoproteins exhibit multifaceted antimicrobial and anti-inflammatory properties that work in tandem with *Lactobacillus*-derived ligands [38,44]. This synergistic ‘Multi-Kingdom’ approach—supported by multi-omics insights into skin-microbiota interactions [19,20]—shifts the therapeutic focus toward active metabolic remodeling, ensuring that the ‘outside-in’ repair is not only rapid but also energetically sustainable for the compromised skin barrier [62,80].

From a methodological standpoint, models that are trained on standardized interventions and well-defined inputs are necessary for forecasting postbiotic efficacy. Baseline clinical phenotype and severity, flare frequency, prior therapy exposure, and objective longitudinal readouts (such as digital flare signals or AI-assisted severity scores) are examples of candidate feature sets. Taxonomic composition, *S. aureus* dominance proxies, and functional signatures are examples of microbiome-related features that, when available, can enhance discrimination. However, this is only possible if data collection is consistent and validated across cohorts. Because they can simulate intricate relationships between clinical variables and high-dimensional biological signals, graph-based or attention-based architectures are particularly appealing in this context. Nevertheless, prospective validation and fairness assessment are still necessary to prevent overfitting and guarantee generalizability across patient subgroups [90,92].

AI-driven prediction is likely to be clinically beneficial when combined with standardized postbiotic products and uniform outcome measures, facilitating reproducible, personalized adjunctive care [90].

The future of AD management lies in the convergence of biotechnology and digital health. A comprehensive overview of how AI facilitates the selection of bioactive postbiotic metabolites and predicts their long-term efficency is presented in Table 4, highlighting the roadmap towards “Smart Postbiotics”.

## 8. Conclusions

The management of AD is undergoing a paradigm shift from reactive, symptom-based treatments to proactive, molecular-targeted interventions. This review has demonstrated that the synergistic integration of *Perilla frutescens*-derived exosomes, *Lactobacillus* postbiotics, and *Saccharomyces ferment* offers a biomimetic, multi-kingdom approach to skin barrier restoration. By simultaneously modulating the JAK–STAT signaling axis, activating the AhR, and promoting lipid homeostasis via PPAR-γ signaling, the PEC addresses the immunological, microbial, and metabolic roots of the disease [11,12,53,76].

Experimental data demonstrating a significant upregulation of elastin (87.9%) and Type I collagen (61.4%) synthesis validate the efficacy of this exosomal delivery system in enhancing skin structural integrity. The addition of *Saccharomyces*-derived peptides and β-glucans further augments this restorative process by providing the metabolic substrates required for the endogenous synthesis of filaggrin and loricrin [38,44,80]. Furthermore, the ability of these bacterial and fungal postbiotics to restore the skin’s physiological pH and mitigate *S. aureus* quorum sensing provides an ecologically precise alternative to conventional antimicrobial agents [17,61,94]. As the demand for ‘steroid-sparing’ therapies grows, this multi-component exosomal system represents a promising frontier in personalized dermatology, offering high biocompatibility and measurable clinical improvements for patients with chronic inflammatory skin disorders [43,62,74].

## 9. Materials and Methods

### 9.1. In Vitro Elastin Synthesis Assay

Human Dermal Fibroblasts (HDF) were cultured and treated with the PEC at concentrations of 2.0% and 5.0% for 24 h. Dexamethasone (DEX) was used as a positive control. Following incubation, intracellular elastin levels were quantified using an ELISA protocol. Results were normalized to the untreated control and expressed as a percentage of control synthesis (*n* = 3).

### 9.2. In Vitro Type I Collagen Evaluation

The effect of the PEC on Type I collagen production was evaluated using HDF cells. The cells were incubated with PEC at 0.01% and 0.05% active concentrations (corresponding to 2.0% and 5.0% use levels) for 24 h. L-ascorbic acid 2-glucoside (AA2G) served as the positive control. Collagen levels were determined using the Sirius Red/Fast Green colorimetric assay, which allows for the simultaneous and selective quantification of collagen and non-collagenous proteins. Absorbance was measured, and the percent increase relative to the untreated control was calculated.

### 9.3. Statistical Analysis

All experiments were performed in triplicate (*n* = 3). Data are presented as mean ± standard deviation (SD). Statistical significance was analyzed using one-way ANOVA followed by post hoc tests, with *p*-values < 0.05 considered statistically significant.

## Figures and Tables

**Table 1:** Comparative Pharmacological Profile and Molecular Targets of Bacterial (Lactobacillus) vs. Fungal (*Saccharomyces*) Postbiotics.

Category	*Lactobacillus* (Bacterial Postbiotics)	*Saccharomyces* (Fungal Postbiotics)	Synergistic Effect (Multi-Kingdom Approach)
Structural Components	Peptidoglycans (PGN), lipoteichoic acids (LTA) [6,24]	β-1,3/1,6-glucans, mannoproteins [25,26]	Complementary structural signaling
Primary Receptors	TLR-2 activation [6,24]	Dectin-1 activation [25,26]	Dual PRR activation
Signaling Pathways	NF-κB, MAPK pathways [6,24]	Dectin-1 and AhR pathways [4,17,20,27]	TLR + JAK–STAT + AhR modulation [12]
Immune Effects	AMP induction (β-defensin, LL-37); immune priming [6,24]	Reduction of Th2 inflammation [25,26]	↓ IL-6 (~40%) [4,8]
Metabolic Activity	Lactic acid (AHA); pH regulation (4.5–5.5) [28,29]	Vitamins (B-complex), amino acids, metabolites [20,27]	Restored acid mantle + metabolic support
Barrier Function	Protease inhibition; antimicrobial barrier support [28,29]	FLG expression → NMF → ↓ TEWL [4,17,20,27]	Enhanced barrier integrity
Oxidative Stress Modulation	Indirect immune-mediated reduction	Antioxidant effect (↓ ROS, ↓ LPO); CYP1A1 modulation [30,31]	Restored redox homeostasis
Microbiome Interaction	Quorum quenching; inhibits *S. aureus* biofilm [32]	Supports microbial balance	Restores eubiosis [15,18,33,34,35]
Cellular Effects	AMP production; pathogen exclusion [36,37]	Keratinocyte proliferation; collagen & loricrin synthesis [38]	Immune defense + tissue repair
Dermal Remodeling	Limited ECM stimulation	↑ Collagen (+11%), ↑ Elastin (+87%) [36,39,40]	Enhanced ECM regeneration
Clinical Outcomes	Reduced infection and inflammation	Improved hydration and barrier repair	↓ erythema, improved skin texture (VISIA) [41,42]
Pharmacological Role	Innate immune activation	Trophic and structural support	Holistic AD management [15,18,33,34,35]

**Abbreviations**: AHA, alpha-hydroxy acid; AhR, aryl hydrocarbon receptor; AMP, antimicrobial peptide; ATP, adenosine triphosphate; CYP1A1, cytochrome P450 1A1; ECM, extracellular matrix; FLG, filaggrin; IL, interleukin; JAK, Janus kinase; LPO, lipid peroxidation; LTA, lipoteichoic acid; MAPK, mitogen-activated protein kinase; NF-κB, nuclear factor kappa B; NMF, natural moisturizing factor; PGN, peptidoglycan; PRR, pattern recognition receptor; ROS, reactive oxygen species; STAT, signal transducer and activator of transcription; TEWL, transepidermal water loss; TLR, Toll-like receptor; Th2, T helper type 2, ↑ denote upregulation/increase, and ↓ denote downregulation/decrease.

**Table 3:** Comparative analysis of exosomal postbiotics vs. conventional therapies in AD management.

Criterion	Exosomal Postbiotics (PEC)	Antibiotics/Antiseptics	Corticosteroids (TCS)
Mechanism of action	Interfere with *agr*-mediated quorum sensing of *S. aureus*, reducing virulence without bactericidal pressure [11,70]	Bactericidal or antiseptic action eliminating bacteria, including commensals [51]	Broad anti-inflammatory and immunosuppressive effects via cytokine inhibition
Target specificity	High; modulates pathogenic behavior while preserving commensals	Low; non-selective microbial elimination	Low; non-specific immune suppression
Selective pressure/resistance	No selective pressure; minimal resistance risk [51]	High selective pressure promoting antimicrobial resistance [51]	No microbial resistance; risk of tachyphylaxis
Impact on microbiome	Preserves commensal microbiota and microbial balance [51,52]	Disrupts microbiome, inducing dysbiosis [47]	Indirect alteration via immune suppression
Effect on biofilms and toxins	Inhibits biofilm formation and dermonecrotic toxin secretion [47]	Limited efficacy against biofilms	No direct antimicrobial effect
Inflammation control	Indirect; immune modulation (e.g., macrophage polarization, JAK–STAT inhibition via luteolin) [63,67]	Limited; reduces pathogen-driven inflammation	Strong and rapid anti-inflammatory effect
Skin barrier/homeostasis	Actively restores barrier and supports physiological signaling [58]	May disrupt skin homeostasis [47]	Improves symptoms without restoring barrier function
Re-colonization risk	Low; supports microbiome-mediated protection [41,52]	High; frequent recurrence after treatment	No effect on microbial recolonization
Safety profile	High; no atrophy or rebound effects reported	Risk of resistance and microbiome disruption	Risk of skin atrophy, rebound flares, and poor adherence (“steroid phobia”) [52]
Therapeutic approach	Proactive, microbiome-oriented, precision medicine-based [54]	Reactive, pathogen-elimination approach	Reactive, flare-control strategy

**Abbreviations**: AD, atopic dermatitis; TCS, topical corticosteroids; *S. aureus*, *Staphylococcus aureus*; agr: accessory gene regulator.

**Table 4:** Integration of AI in predicting postbiotic efficacy in AD.

Domain	AI Application	Key Features/Inputs	Clinical Relevance
Predictive modeling	Risk prediction and treatment response estimation	Baseline severity, flare history, treatment exposure, longitudinal barrier and microbiome data	Enables personalized prediction of flare risk and likelihood of response to postbiotics [90,91]
Disease stratification	Machine learning-based deep phenotyping	Clinical phenotype, disease trajectory patterns, multi-omics data	Identifies patient subgroups with distinct disease trajectories and therapeutic needs [93]
Severity assessment	AI-assisted or automated scoring systems	Standardized severity indices, imaging, longitudinal clinical data	Reduces inter-observer variability and improves monitoring consistency [93]
Digital biomarkers	Passive flare detection and monitoring	Wearable/device-derived signals, longitudinal symptom tracking	Enables early detection of flares and proactive therapeutic adjustments [92,93]
Treatment optimization	AI-guided stratification linked to treatment pathways	Integrated datasets (clinical, microbiome, omics)	Supports precision medicine and individualized postbiotic regimens [92]
Hybrid therapeutic integration	AI-supported “inside-out” and “outside-in” approaches	Clinical response to biologics + barrier restoration markers (e.g., filaggrin, loricrin)	Enhances synergy between systemic biologics and topical postbiotics; supports steroid-sparing strategies
Mechanistic targeting	Modeling of pathway-specific effects	AhR activation, cytokine profiles, oxidative stress pathways (e.g., CYP1A1)	Predicts response to postbiotics targeting barrier repair and immune modulation
Model architecture	Advanced AI frameworks	Graph-based models, attention-based architectures	Captures complex relationships between clinical and biological variables [88,92]
Data requirements	Standardized and validated datasets	Clinical variables, microbiome composition, *S. aureus* dominance, functional signatures	Ensures reproducibility and generalizability across patient populations
Limitations & considerations	Validation and ethical use	External validation, fairness assessment, transparent inputs	Prevents bias, overfitting, and ensures clinical applicability [90,91]
Clinical implementation	Decision-support systems	Integrated patient data and standardized interventions	Supports clinicians without replacing judgment; improves personalized adjunctive care [90]

**Abbreviations**: AD, atopic dermatitis; AI, artificial intelligence; AhR, aryl hydrocarbon receptor; CYP1A1, cytochrome P450 1A1.

## Data Availability

No new data were created or analyzed in this study. Data sharing is not applicable to this article.

## Abbreviations

The following abbreviations are used in this manuscript:

AA2G	Ascorbyl Glucoside
AD	Atopic Dermatitis
Agr	Accessory Gene Regulator
AgrC	Accessory Gene Regulator C
AHA	Alpha-hydroxy acid
AhR	Aryl hydrocarbon receptor
AI	Artificial Intelligence
AIP	Autoinducing Peptides
AMP	Antimicrobial peptide
ANOVA	Analysis of Variance
ATP	Adenosine triphosphate
CCL2/11	Chemokine ligand 2/11
CCR3	Chemokine receptor 3
CIS	Cytokine-Induced Stat
CLDN-1	Claudin-1
CYP1A1	Cytochrome P450 1A1
CXCL14	C-X-C Morif Chemokine Ligand 14
DEX	Dexamethasone
ECM	Extracellular matrix
ELISA	Enzyme-Linked Immunosorbent Assay
EPCR	Endothelial Protein C Receptor
ERK	Extracellular Signal-Regulated Kinase
EVs	Extracellular Vesicles
FLG	Filaggrin
GMP	Good Manufacturing Practice
hBD-2	Human Beta-Defensin 2
hBD-3	Human Beta-Defensin 3
HDF	Human Dermal Fibroblasts
HMGB1	High Mobility Group Box Protein 1
IgE	Immunoglobulin E
IKK- β	Inhibitor of Nuclear Factor Kappa-B Kinase Subunit Beta
IL-4	Interleukine 4
IL-5	Interleukine 5
IL-6	Interleukine 6
IL-8	Interleukine 8
IL-10	Interleukine 10
IL-13	Interleukine 13
IL-17	Interleukine 17
IL-22	Interleukine 22
IL-23	Interleukine 23
IL-31	Interleukine 31
IL-1 α	Interleukine 1α
IL-36 α	Interleukine 36α
ISAPP	International Scientific Association for Probiotics and Prebiotics
JAK 1	Janus kinase 1
JAK 1	Janus kinase 2
JAK–STAT	Janus Kinase–Signal Transducer and Activator of Transcription
LL-37	Cathelicidin Antimicrobial Peptide
LPO	Lipid peroxidation
LTA	Lipoteichoic acid
MAPK	Mitogen-activated protein kinase
MMPs	Matrix Metalloproteinases
NF-κB	Nuclear factor kappa B
NHE1	Sodium-Hydrogen Exchanger 1
NMF	Natural moisturizing factor
NRF2	Nuclear Factor Erythroid 2-Related Factor 2
NTA	Nanoparticle tracking Analysis
0/W	Oil-in-Water
PAMPs	Pathogen-Associated Molecular Patterns
PEC	*Perilla*-derived exosomal complex
PGN	Peptidoglycan
PIA	Polysaccharide Intercellular Adhesin
PPAR-γ	Peroxisome Proliferator-Activated Receptor gamma
PRR	Pattern recognition receptor
PSM α	Phenol-Soluble Modulin alpha
QQ	Quorum Quenching
ROS	Reactive oxygen species
SCORAD	SCORing Atopic Dermatitis
SOCS	Suppressors of Cytokine Signaling
SD	Standard Deviation
STAT 3	Signal transducer and activator of transcription 3
TACE	TNF-alpha Converting Enzyme
TCS	Topical Corticosteroids
TEWL	Transepidermal water loss
TGF-βR	Transforming Growth Factor-beta Receptor
Th2	T Helper Type 2 cells
TLR	Toll-like receptor
TNF- α	Tumor Necrosis Factor-alpha
TSLP	Thymic Stromal Lymphopoietin
TYK2	Tyrosine Kinase 2
